# Signaling Cascades of *Pasteurella multocida* Toxin in Immune Evasion

**DOI:** 10.3390/toxins5091664

**Published:** 2013-09-24

**Authors:** Katharina F. Kubatzky, Bianca Kloos, Dagmar Hildebrand

**Affiliations:** Medical Microbiology and Hygiene, Department of Infectious Diseases, University Hospital Heidelberg, Im Neuenheimer Feld 324, Heidelberg 69120, Germany; E-Mails: bianca.kloos@med.uni-heidelberg.de (B.K.); dagmar.hildebrand@med.uni-heidelberg.de (D.H.)

**Keywords:** bacterial toxin, *Pasteurella multocida*, immune evasion, signal transduction, osteoclast, osteoimmunology, carcinogenesis

## Abstract

*Pasteurella multocida* toxin (PMT) is a protein toxin found in toxigenic strains of *Pasteurella multocida*. PMT is the causative agent for atrophic rhinitis in pigs, a disease characterized by loss of nasal turbinate bones due to an inhibition of osteoblast function and an increase in osteoclast activity and numbers. Apart from this, PMT acts as a strong mitogen, protects from apoptosis and has an impact on the differentiation and function of immune cells. Many signaling pathways have been elucidated, however, the effect of these signaling cascades as a means to subvert the host’s immune system are just beginning to unravel.

## 1. Introduction

*Pasteurella multocida* are Gram-negative bacteria that cause a variety of different disease syndromes in various animals [1]. Depending on the serotype, specific types of diseases are associated with specific hosts. Type D and A strains for example are associated with upper respiratory tract infections in pigs and toxigenic strains expressing the *Pasteurella multocida* Toxin (PMT) cause porcine atrophic rhinitis [2]. This disease is characterized by an atrophy of the nasal ventral conchae bones and a shortening or distortion of the snout [3]. After purification of the toxin in the late 1980s, it was shown that the purified toxin alone was sufficient to induce bone lesions and thus must be the causative agent of porcine atrophic rhinitis [4,5,6]. PMT has therefore been considered as an osteolytic agent that induces osteoclast formation and inhibits osteoblast activity and bone remodeling. In the past few years, signaling pathways initiated by PMT have been investigated in a number of different cellular model systems and it is now becoming clear that PMT might be a weapon of *Pasteurella* bacteria to populate the host and to fight the immune system by different toxin-related immune evasion strategies [7].

To avoid infectious diseases, higher organisms have developed a highly differentiated and adaptable defense machinery in form of the immune system. On the other side pathogens have acquired distinct strategies to avoid recognition and elimination by the host’s immune system in order to survive and replicate. In this context, the modulation of signaling cascades of immune cells represents one effective strategy [8,9,10,11]. In the following paragraphs *Pasteurella multocida* toxin related signaling cascades and their importance are discussed in the context of inflammation and immune evasion as well as their potential benefit for the pathogen.

## 2. Molecular Pathways

**Figure 1:**
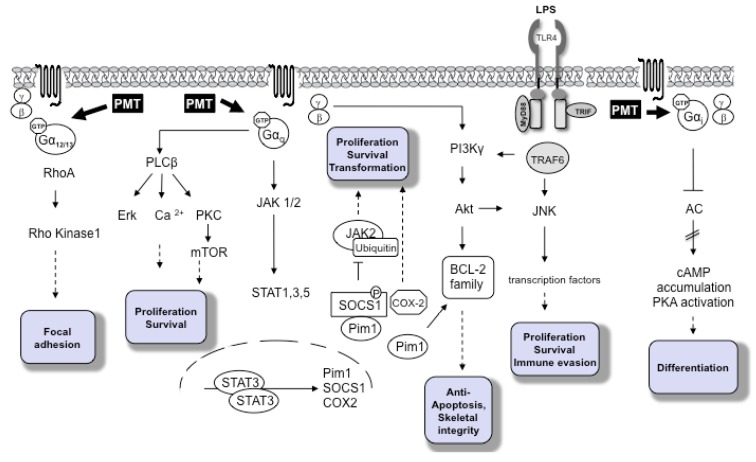
*Pasteurella multocida* toxin (PMT)-dependent signaling pathways. PMT modifies the Gα subunits of the heterotrimeric G proteins Gα_i_, Gα_q/11_ and Gα_12/13_ through deamidation. The major subsequent signaling pathways are depicted according to the activated Gα subunit. The cellular outcomes are described in the shaded boxes.

PMT is a 146 kDa AB protein toxin consisting of an *N*-terminal receptor binding and translocation domain and a *C*-terminal domain harboring the catalytic activity. After binding to a yet unknown surface receptor and receptor-mediated endocytosis, PMT enters the cell and translocates to the cytosol from late endosomes [12]. The substrates of PMT are the alpha-subunits of specific heterotrimeric G proteins, where it deamidates glutamine 205 to glutamic acid via PMT’s thiol protease-like catalytic triad [13]. Using a mass spectrometry and a 2D gel electrophoresis approach, respectively, the substrate specificity of PMT for the members of heterotrimeric G proteins was recently investigated in detail [14,15]. The data show that PMT not only activates Gα_i2_ [16,17], but also the related family members Gα_i1_ and Gα_i3_. In contrast to previous results, both Gα_11_ and Gα_q_ get deamidated [18], as do Gα_12_ and Gα_13_. Gα_s_, however, is not a substrate. It seems that the *N*-terminus of the respective G protein determines the substrate recognition by PMT. Additionally, it is known that for PMT function, the release of the Gβγ subunit is necessary for downstream signaling as mutants of Gα_q_ incapable of binding or releasing Gβγ are not activated by PMT [19]. Downstream of G proteins PMT activates a number of signaling cascades. Release of the βγ subunit from the respective G protein stimulates phosphoinositide 3-kinase (PI3K) γ and results in the production of phosphatidylinositol-3,4,5-trisphosphate (PIP_3_). Downstream of Gα_q_ PMT activates typical G protein related pathways, such as activation of phospholipase C (PLC) β, eventually leading to diacylglycerol (DAG) and inositol triphosphate (IP_3_) production and Ca^2+^ release, whereas downstream of Gα_12/13_ Rho GTPase activation results in cytoskeletal rearrangements. As a consequence of Gα_i_ activation, PMT inhibits stimulation of adenylate cyclase and cAMP production from ATP [17,20]. In addition to the classical G-protein related signaling cascades, other signaling pathways, often with a role in mitogenesis, differentiation or cell survival, are activated (Figure 1). For an excellent overview on this topic see [20].

## 3. PMT as A Potential Carcinogen

### 3.1. Inflammation and Cancer

Acute inflammatory reactions are an important part of the immune response, however, prolonged or aberrant inflammation can lead to a variety of pathologies with cancer being one of them [21,22,23]. Three main mechanisms have been identified to be involved in cancerogenesis through infectious agents: chronic inflammation, cellular transformation or the suppression of the immune system as it is found in HIV-infected cells [24]. The process of cellular transformation can occur for example through integration of the viral genome in a cell, the expression of virally encoded oncogenes or inhibitors of tumor suppressors, respectively. Persistent bacterial infections are therefore discussed to be a cause of cancer [25]. As carcinogenesis is a multistage process that can take years to result in an obvious pathology, it is often difficult to determine the factors that contributed to the development of the disease or that supported the process of cellular transformation. However, there is now increasing evidence that pathogenic bacteria can contribute to specific stages in cancer development, particularly in chronic infections [26]. Especially in the past decade, epidemiologic data have been generated that support the theory that chronic inflammation can be linked to cancer [27,28]. It is also becoming increasingly clear that not only pathogenic bacteria but also commensal bacteria that are part of the microbiota can cause inflammation-associated carcinogenesis, if they leave their microenvironment and invade the skin or mucosa [29]. In addition, bacterial protein toxins that modify cell signaling pathways of the host directly, cause aberrant signaling leading to inflammation and tumorigenesis [26,30,31]. Inflammatory signaling is characterized by the production of cytokines such as Interleukin (IL-)1, IL-8 and tumor necrosis factor (TNF) α, an increase in the expression of adhesion factors, cyclooxygenase (COX)2-mediated production of prostaglandins and oxidative and nitrative stress [25]. These pathways can be activated via pattern recognition receptors, such as toll like receptors (TLR) that sense the presence of microbial products. TLR signaling results in the activation of the transcription factor NFκB and of the MAP kinase pathways, which play a pivotal role in the induction of pro-inflammatory signaling and the subsequent release of inflammatory mediators. In the case of bacterial protein toxins these signaling pathways are often targeted directly by the bacterial compound, for example through enzymatical modification by bacterial AB toxins which constitutively perturbs cellular signaling processes. Ultimately, such signaling events can culminate in direct DNA damage, inhibition of apoptosis, cytoskeletal changes, stimulation of proliferation or inhibition of cell cycle progression, increased angiogenesis and suppression of an adequate immune response [32]. Taken together, chronic infections with bacteria that perturb cell signaling processes may be able to contribute to cellular transformation by generating an environment that is characterized by increased cell survival, proliferation and aberrant immune cell signaling which ultimately promotes tumor initiation and promotion [25,26].

### 3.2. PMT and Cancer

**Figure 2:**
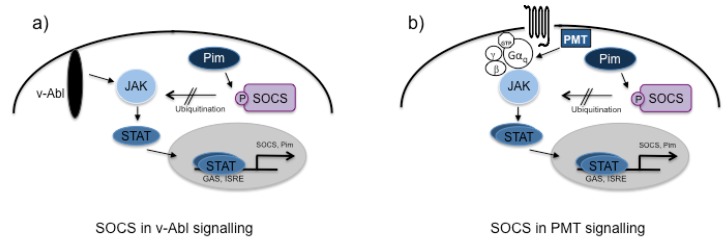
Pim-dependent inactivation of the negative regulator SOCS. (**a**) In v-Abl as well as in (**b**) PMT-mediated signaling, activation of the JAK-STAT pathway leads to the constitutive expression of the otherwise short-lived serine/threonine kinase Pim. Phosphorylation of its downstream target, the suppressor of cytokine signaling (SOCS) inhibits the ability of SOCS to act as an ubiquitin ligase. Thus JAK molecules accumulate in the cell leading to the hyperactivation of STAT3 signaling, increased cell survival and anchorage-independent growth.

Very early, PMT was found to be a potent mitogen for a number of different cell types [33,34,35]. Thus it was suggested by some authors that PMT might be able to act as a carcinogen [26,30,31,32,36]. PMT is known to activate mitogenic signaling cascades such as MAP kinases and the JAK-STAT pathways, which are often found to be aberrantly activated in cancer [20,37,38]. The negative regulation of cytokine signaling and the downstream activation of the JAK-STAT pathway is a crucial part of the immune response in order to avoid excessive inflammation that could result in cell death and tissue damage. Well known regulators of cytokine responses are the suppressors of cytokine signaling (SOCS). The members of this family (SOCS1–SOCS7 and CIS) are induced through the cytokine-receptor-mediated JAK-STAT cascade and they act as negative feedback inhibitors [39]. Their inhibitory function can be transduced through direct binding to Janus kinases or cytokine-receptors, competition with STAT proteins for phosphorylated binding sites at the receptor or through E3-Ligase-mediated degradation of cascade constituents [39]*.* As a consequence, cytokine-stimulated JAK-STAT activation is usually short-lived. In contrast to these results, we could show that the PMT-induced activation of this pathway lasted for over 18 h as the expression of the STAT-dependent SOCS gene was suppressed [37]. Astonishingly, in contrast to what had previously been reported for cytokine-induced pathways, overexpression of SOCS1 even potentiated the activation of PMT-induced STAT3. This was also the case when PMT-treated cells were co-stimulated with a TLR ligand, leading to the expression of endogenous SOCS1, as it would happen in a cell during a naturally occurring infection with *Pasteurella multocida*. Responsible for this hyper-activation is the PMT-induced, STAT-dependent serine/threonine kinase Pim-1, which mediates serine/threonine phosphorylation of SOCS1 [40]. Because phosphorylated SOCS1 cannot interact with JAK2 to tag it for proteasomal degradation, JAK2 accumulates in the cell, which eventually causes hyper-activation of its target, the transcription factor STAT3 [40] (see Figure 2). Similarly to NFκB, STAT3 has been shown to act as a non-classical oncogene and plays a role in inflammation-associated cancer underlining its importance in a potential role for PMT in cancer [41]. Interestingly, the viral kinase v-Abl. which is encoded by the Abelson murine leukemia virus, uses this pathway to trigger cell survival and to generate an environment where the virus can replicate. It was found that the v-Abl-dependent JAK activation was required for cytokine-independent growth, tumor formation in a nude mice model and transformation of bone marrow cells and it was hypothesised that hijacking the SOCS regulatory pathway is indispensable for cellular transformation [42,43]. The fact that PMT also uses this evasion strategy suggests that it must be useful for the bacteria in some way. *Pasteurella multocida* is not a typical intracellular pathogen, however, the ability to invade host cells has been described in several publications [44,45,46,47,48,49,50]. However, whether toxigenic strains have an advantage in internalization or intracellular survival has not been studied yet.

Pim-1 is a well known survival-kinase [51]*.* Additionally, PMT also activates another survival kinase, the serine/threonine kinase Akt, an effector-kinase of PI3Kγ, through liberation of the βγ subunit from the respective Gα subunit [19]. We demonstrated that PMT-stimulated Akt and Pim-1 cooperate in the regulation of pro- and anti-apoptotic members of the Bcl-2 (B-cell lymphoma/leukaemia 2) family and thereby protect HEK293 cells, lung cancer cells and melanoma from staurosporine- and chemotherapeutic-initiated apoptosis, respectively [52]. Activation of these survival pathways could be beneficial for the bacterium for two reasons. First of all, Bcl-2 is essential for the differentiation, survival and activity of osteoclasts and has a determining part in maintaining skeletal integrity [53,54]. This fits well with the known impact of PMT on bone-degradation and we will later review the connection of the bone and the immune system in more detail. Secondly, Bcl-2 has a negative impact on the inflammasome as a crucial component of the innate immunity. It binds directly to NALP1 of the multi-protein complex inflammasome and prevents the activation of Caspase-1 [55,56]. As Caspase-1 is required to activate pro-IL-1β into mature IL-1β, Bcl-2 plays a central role in the regulation of immune and inflammatory responses to infections.

Recently it was found that the mammalian target of rapamycin (mTOR) is activated by PMT [57]. Dysregulation of mTOR, for example via excessive activation of the PI3K pathway, mediates the progression of cancer [58]. Additionally, mTOR is involved in persistent inflammation [59]. While mTORC1 plays a role in protein translation, cell growth, proliferation and autophagy, mTORC2 is supposed to be important for actin cytoskeletal reorganization [60]. Despite the significant impact that PMT has on the cytoskeleton through activation of the Rho GTPase RhoA, only mTORC1 was found to be activated by PMT. Although mTOR is activated via Gα_q_-dependent activation of PKC, this activation is not only directly mediated by PMT, but is increased via an auto/paracrine stimulation through factors secreted into the cell supernatant [57,61]. Connective tissue growth factor (CTGF), a matricellular protein of the CCN family, was found to be upregulated in PMT-treated cells. Interestingly, CTGF plays a role in cancer and inflammation as well as osteogenesis [62]. These findings suggest that it will be of interest to perform a proteome analysis of supernatants from PMT-treated cells from various cell lines and under different conditions to get a more comprehensive understanding of PMT’s ability to directly and indirectly trigger signaling pathways involved in oncogenic signaling.

Another characteristic process in the development of cancer through infectious agents is the creation of a persistent inflammatory response through cells of the immune system. While the original publications on the toxigenic infections or PMT treatment of animals provided no evidence of an inflammatory process, other animal models showed that PMT treatment causes inflammation [63,64]. In addition, our *in vitro* data using macrophage cell lines or primary bone marrow-derived immune cells show that PMT triggers the production of various pro-inflammatory cytokines [65,66]. Often the production of these cytokines is associated with increased cellular proliferation and can thus impact on the initiation or progression of tumor growth; whether there is also a growth advantage for the pathogen, has not been investigated yet for *Pasteurella multocida*.

Due to its interesting signaling properties, *Pasteurella multocida* Toxin has been discussed as another paradigm of a carcinogenic bacterial protein toxin [26,30,31,32,36]. However, no data are available whether all these *in vitro* findings do have relevance *in vivo*. Furthermore, it is unclear whether the effects of PMT on cell proliferation, inhibition of apoptosis and anchorage-independent growth are only transient, *i.e.*, in the presence of toxin, or whether the cell is actually transformed. To investigate this, the effect of chronic exposure of cells to PMT has to be investigated where the long-term epigenetic changes will be monitored and evaluated.

## 4. PMT-Mediated Pathways in Immune Cells

Although most data regarding PMT-induced signaling cascades have been generated using cell lines such as HEK293 or Swiss3T3 cells to investigate the cellular action of PMT, some publications also discuss the impact of PMT on immune cells. As we discussed before, STAT3 is one of the transcription factors that is constitutively activated by PMT [37]. While this leads to the protection from apoptosis, increased survival and anchorage-independent growth, STAT3 is also known to be a negative regulator of the immune response. STAT3 directs the development of antigen presenting cells (APCs) towards a tolerogenic, *i.e.*, a tolerance-inducing, phenotype [67]. As APCs represent a pivotal first barrier against microbes and also interact with adaptive immune cells, their signal transduction pathways represent an important target for microbial organisms. Using human monocytes, we found that PMT modulates the immune reactions of these APCs. Within the host, Gram-negative bacteria such as *Pasteurella multocida* are typically sensed by their cell wall constituent lipopolysaccharide (LPS) that binds in complex with the LPS-binding protein to a complex of TLR4, CD14 and the associated protein MD-2 [68]*.* The TLR4-mediated signaling cascades induce the expression of a variety of pro-inflammatory cytokines and costimulatory molecules on the APC [69,70]. A variety of Gram-negative bacteria are known to influence TLR4 signaling pathways through the activation of heterotrimeric G proteins via bacterial protein toxins. Examples include Cholera Toxin (CT) of *Vibrio cholerae*, heat-labile enterotoxin (LT) of enteropathic *Escherichia coli* or Pertussis Toxin (Ptx) of *Bordetella pertussis*, which act on heterotrimeric G proteins and thus alter LPS-induced gene expression. This manipulation is mediated by a change in the production of cAMP through the G proteins and a subsequent shift of cytokine expression in the APC [71,72]. PMT also modulates LPS-stimulated surface protein and cytokine production and completely suppresses TLR4-induced IL-12p40 production [66,73]. In the proposed model, the TLR4-mediated, NFĸB-induced production of IL-12p40 is suppressed by Gα_i_-mediated inhibition of adenylate cyclase and cAMP accumulation and by Gβγ-mediated activation of PI3kinase and JNK activation. The bioactive heterodimer IL-12p70, consisting of covalently bound p35 and p40 [74]*,* does not only play a direct role in T helper cell differentiation but also displays a strong synergistic effect with the B7/CD28 interaction of APCs and T cells needed to induce T cell proliferation [75,76]. Indeed the PMT-induced abrogation of IL-12 expression prevents APC-mediated T cell proliferation. Therefore, by targeting TLR4-mediated signaling, PMT impairs inflammation and eventually the activation of T cells. However, PMT alone does not activate monocytes or DCs derived from human monocytes (MDDCs) [66,73]. Only when the pathogen is sensed by the host cell through LPS-mediated TLR4 activation and defense cascades are initiated leading to the activation of APCs, the toxin manipulates the outcome of the signaling and thereby represses the immune response.

Apart from the modulated production of cytokines, Blöcker *et al.* suggest that the activation of the cytoskeleton through PMT-mediated RhoA activation is a means to change shape and thus function of DCs [77]. Due to the PMT-mediated irreversibly enhanced actin polymerization, the ability of the cells to migrate in response to a chemokine stimulus was downregulated. As the migration of immature DCs to the peripheral sites in order to react to and process antigens and to subsequently present them to T cells is a prerequisite in initiating an immune response, targeting of the cytoskeletal system is actually a common strategy of bacterial protein toxins to subvert the immune system [78,79,80]. Thus, interference with the migrational potential of DCs is an effective mechanism to limit T cell responses and an efficient mechanism to reduce affinity-matured humoral responses by B cells which offers the pathogen a survival advantage.

Interestingly, PMT not only affects cells of the innate immune system, but it can also modulate B cells, that are part of the adaptive immune system, in a direct manner [65]. The stimulation of murine bone marrow cells with PMT generated two cell populations that were able to survive with PMT. These were characterized to be B cells and macrophages. This selective survival indicated that these two cell populations are two main targets of the toxin, since PMT was found to be taken up by all other haematopoietic cells tested [7]. As PMT can selectively influence B cells, we suggest that it interferes with their differentiation fate by changing intracellular signaling events, which could result in a survival advantage of the *Pasteurella multocida* bacteria in the sense of immune evasion. Additionally, the data showed that PMT-treated B cells are important to efficiently drive osteoclast differentiation from macrophages by secretion of inflammatory and osteogenic cytokines such as receptor activator nuclear factor kappa B ligand (RANKL), IL-1β, IL-6 and TNF-α.

Interestingly, it was reported very early from several groups that PMT does not seem to be a good adjuvant *in vivo* as the antibody production in response to oral immunization with PMT and ovalbumin was suppressed. Additionally, PMT also reduced the adjuvant effect of other toxins like the Cholera Toxin on ovalbumin (OVA) immunization, suggesting that the toxin actively modulates B cells to prevent correct antibody production [73,81]. However, after heat-inactivation, through the introduction of point mutations inactivating the catalytic domain or by chemical treatment of the protein, the immunogenicity of PMT can be increased [82,83,84]. This suggests that the toxin in its native form stimulates B cell signaling in a way that prevents the specific activation of the adaptive immune system which would result in a survival advantage. This could for example occur through inhibition of the affinity maturation of the B cells, but this has to be investigated yet.

**Figure 3:**
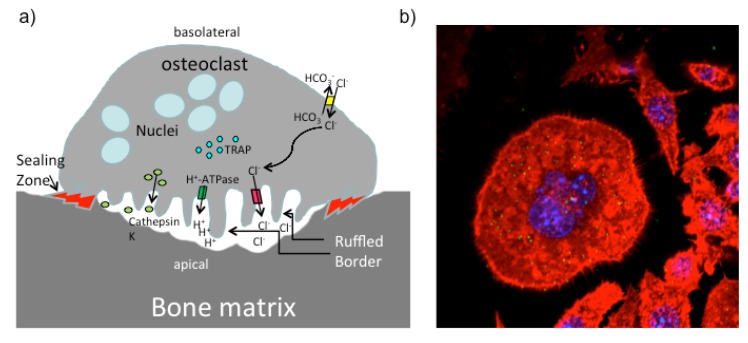
Illustration of a functional active osteoclast. (**a**) Mature osteoclasts are large multi-nucleated cells that cover a big area on the bone to degrade the bone matrix. The apical membrane faces the bone and the sealing zone generates an isolated region. A ring of aggregated F-actin assures the strong attachment of the osteoclast to its substrate. The resorptive area is acidified by secretion of HCL to demineralize the bone matrix. Organic components are degraded by Cathepsin K. Osteoclasts express tartrate-resistant acid phosphatase (TRAP), which is commonly used as a marker for osteoclasts. (**b**) Osteoclast derived from PMT-treated RAW 264.7 macrophages. RAW 264.7 cells were stimulated with PMT for 4 days and osteoclasts were identified by staining for multi-nucleated and TRAP-positive cells (blue: nuclei (DAPI); green: TRAP enzyme (ELF97); red: cytoskeleton (Phalloidin-TRITC)).

## 5. PMT and the Skeletal System

It has recently been appreciated that there is a close connection between the immune system and the skeletal system which led to the definition of a new research area dedicated to this topic, the field of osteoimmunology [85]. Bone homeostasis is achieved by an equilibrium between bone build-up through osteoblasts and bone-degradation performed by osteoclasts, as bone-resorbing cells. Osteoblasts differentiate from mesenchymal cells while the progenitors for osteoclasts are haematopoietic cells of the myeloid lineage. The differentiation process of osteoclasts from monocytes or macrophage progenitor cells is initiated by signals through the cytokine RANKL. During differentiation cells fuse into big, multi-nucleated cells that express the necessary repertoire of proteins needed to establish their ruffled border and a sealing zone that is characterized by accumulation of F-actin and a strong attachment which is important to safely resorb bone in a controlled fashion on the apical side of the polar osteoclast [86] (Figure 3). Although PMT had been identified because of its osteolytic function in atrophic rhinitis, the mechanism how PMT interferes with bone homeostasis is just beginning to be elucidated [6,87]. Research from the various labs working with PMT however clearly suggests that PMT triggers bone degradation by modulating the differentiation and activity of both cell types, osteoclasts as well as osteoblasts.

### 5.1. PMT and Osteoclasts

Once PMT had been identified as the causative virulence factor of atrophic rhinitis, *in vivo* studies investigated the effect of PMT in animals. In a rat model, it was for example shown that intra-peritoneally injected PMT affected femoral and nasal turbinate bones in rats by decreasing the size of the nasal turbinate bones [35]. Osteoclasts found in sections of these PMT-treated rats displayed an increase in cell size compared to control untreated rats, contained huge vacuoles within the cytoplasm and had an intensive ruffled border upon PMT treatment. In addition, PMT-generated osteoclasts were tartrate resistant acid phosphatase (TRAP)-positive and multinucleated cells. In the following years, *in vitro* setups were used that allowed to cultivate specific cell populations that could be studied in greater detail. Using a non-adherent cell population from murine peripheral blood mononuclear cells (PBMCs), Jutras *et al.* showed that stimulation with PMT generated many TRAP-positive mononuclear osteoclast progenitor cells and also stimulated the terminal differentiation of these cells into osteoclasts [88]. In another setup, mononuclear bone marrow cells isolated from bones of pigs were cultivated in medium containing 1,25-Dihydroxy Vitamin D_3_ as a stimulator of osteoclast formation and PMT, respectively, which induced the differentiation of TRAP-positive multi-nucleated cells [89]. However, cells differentiated with PMT stained less positive for TRAP, although they appeared to be functional and did not display an obvious defect in osteoclast resorption on bovine slices. As discussed before, PMT might sometimes act through the induction of the secretion of soluble factors that in this scenario could cause increased differentiation or decrease the production of osteoclastogenesis-inhibiting factors. Indeed, the analysis of supernatants from PMT-treated progenitor cells indicated that PMT acts via the secretion of soluble mediators to trigger osteoclastogenesis [89].

Besides the impact of PMT on osteoclast differentiation, it was shown that PMT activates the resorption capacity of isolated mature osteoclasts on bone cubes cut from pig-derived long bones [90]. PMT did not increase the resorption capacity of sorted mature osteoclasts directly, but osteoclasts had to be cultivated together with osteoblasts allowing direct cell-cell contact while the mere exchange of soluble factors between the two populations was insufficient for this effect [90]. However, none of these data explained the observed phenotype of increased bone loss mechanistically.

In addition to the result that osteoblasts seem to play an important role for PMT to mediate its effects on osteoclast differentiation and function, we could recently show that B cells are needed to efficiently stimulate osteoclast differentiation with PMT using isolated cell populations from bone marrow of mouse [65]. The data showed that osteoclastogenesis is most efficient when isolated macrophages are stimulated together with B cells in a co-culture system. The exact mechanism of this interaction is far from being understood, but it is clear that secreted cytokines are involved in that process. We hypothesize that the dramatic effect of PMT on osteoclast differentiation occurs as a collateral damage when PMT interferes with the immune system to subvert it for survival purposes. In addition, the microorganism obviously also benefits from the differentiation of macrophages into osteoclasts, as this reduces the number of phagocytic cells.

### 5.2. PMT and Osteoblasts

Besides directly targeting bone degradation via interference with osteoclast differentiation and activity, it is known that PMT also acts on osteoblasts to inhibit their bone-building capacity. The stimulation of an osteoblastic cell line ROS 17/2.8 with PMT resulted in the down-regulation of expression of the alkaline phosphatase (ALP), an enzyme required for setting-up mineralized bone nodules by the osteoblasts [91]. These findings could then be extended to primary embryonic chicken-derived osteoblasts, where PMT was found to act as a mitogen and to down-regulate the expression of ALP [90,92]. Taken together these data point to a reduction of bone formation due to a diminished differentiation into fully functional osteoblasts with PMT.

Recently, the stromal cell line ST-2 was used to investigate whether PMT acts also on the osteoblast differentiation in addition to its effect on osteoblast activity [93]. ST-2 cells can differentiate into adipocytes and osteoblasts, making them a good model system to investigate the effect of PMT on osteoblastic progenitor cells. PMT sufficiently blocked osteoblast differentiation but not adipocyte differentiation. However, PMT increased the number of adipocytes. Although this has not been investigated in this context yet, it is intriguing to hypothesize that these adipocytes might present yet another link to persistent inflammation [94,95]. Mechanistically, PMT was found to down-regulate the expression of two transcription factors, Runx2 and Osterix [96], that are involved in osteoblastogenesis, eventually causing the downregulation of ALP leading to a lack of mineralization nodules [93]. The major pathway downstream of PMT-activated Gα_q_ and Gα_12/13_ is the small GTPase RhoA [97]. Since RhoA had previously been shown to inhibit osteoblast formation [98], the effect of RhoA on osteoblast activity was investigated in more detail. While constitutive RhoA activation by PMT led to an inhibition of osteoblastogenesis, inhibition of the downstream effector kinase of RhoA, ROCK, reversed the effect and even stimulated osteoblast formation [93].

This ability of PMT to interfere with the differentiating fates of osteoblasts could happen coincidently as a side-effect while PMT is primarily targeting other cells, such as immune cells. However, it is also possible that direct modulation of bone build-up is part of the immune evasion strategies that *Pasteurella multocida* has developed to achieve optimal growth conditions within the host. Here we can only come up with creative ideas as to how this could be beneficial for the bacteria. One possible scenario is that PMT diminishes osteoblast activity and stimulates osteoclast function, respectively, to generate a niche within the bone to hide and replicate. Another speculation is that PMT might be able to dedifferentiate osteoblasts into progenitor cells. There is literature available that suggests that fibroblasts, mesenchymal stem cells or stromal cells have the ability to suppress inflammation under certain conditions [99,100,101]. Although this is merely speculation, it is an intriguing idea that PMT could actively generate an anti-inflammatory microenvironment.

## 6. Concluding Remarks

In order to effectively colonize a host and to replicate inside host cells or attached to the cell surface, microorganisms have evolved complex strategies to successfully adapt to their host and to avoid recognition. While the immune system of the host tries to eradicate the microbial pathogen through inflammation, the pathogen itself has various defense mechanisms ensuring its survival [10].

Classically, PMT has been known as the causative agent of atrophic rhinitis in pigs. Therefore, it was assumed that its main function is to act as an osteolytic agent. However, it is unlikely that bacteria such as *Pasteurella multocida* would have an advantage by actively degrading bone and thus would dedicate the function of a bacterial protein toxin to this purpose. With a much better understanding of the molecular pathways of PMT and with the use of different *in vitro* model systems it is now becoming clear that the effect of PMT on bone homeostasis is just one strategy of this microorganism to evade the host’s immune response. Among PMT’s other escape mechanisms are typical anti-immune strategies such as to hide from immune-surveillance through manipulation of macrophage activity via an increase in osteoclast differentiation, the blockage of acquired immunity through the manipulation of B cells, down-regulation of apoptosis, interference with TLR signaling which dampens the immune response and the manipulation of intrinsic cellular pathways to change the transcriptional program of a given cell type [11]. All these strategies increase the chance of survival for the microorganism. Obviously, there is a homeostatic regulation between the various defense mechanisms of the host and the microbe and the fact that PMT induces the production of pro-inflammatory cytokines does not mean that the host ultimately wins the battle between the two. Rather, this microbial-triggered inflammation can cause secondary damage. In contrast to normal inflammation that is self-limiting, persistent presence of PMT could cause chronic inflammation due to the failure of the immune system to resolve the inflammatory response. The effects of PMT on the various cells of the immune system and other parts of the body are just beginning to get resolved and with our increasing understanding of the molecular pathways targeted by PMT we will better understand the intricate interplay between the immune system and bacterial infections.
